# The Role of Body Mass Index and Waist Circumference in Gender-Specific Risk Factors for Stress Urinary Incontinence: A Cross-Sectional Study

**DOI:** 10.7759/cureus.38917

**Published:** 2023-05-12

**Authors:** Marc Ganz, Christopher Alessandro, Menachem Jacobs, Daniel Miller, Jonathan Diah, Andrew Winer, John M Shields, Jeffrey Weiss

**Affiliations:** 1 Public Health Sciences, State University of New York Downstate Health Sciences University, Brooklyn, USA; 2 Internal Medicine, Icahn School of Medicine at Mount Sinai, Queens Hospital Center, Queens, USA; 3 Urology, State University of New York Downstate Health Sciences University, Brooklyn, USA; 4 Urology, Kings County Hospital Center, Brooklyn, USA

**Keywords:** body mass index (bmi), family medicine, waist circumference, urology, stress urinary incontinence

## Abstract

Background

Urinary incontinence is the loss of bladder control and is a common condition found more often in women. Incontinence can present in several ways. The various forms of incontinence include urgency urinary incontinence, stress urinary incontinence, and mixed urinary incontinence (a combination of both stress urinary incontinence and urgency urinary incontinence).

Studies have been conflicting on the prevalence of UI in obese women compared to non-obese women. The subtypes of incontinence may play a role in the discrepancy currently found in research. In addition to the discrepancy seen between subtypes, there may be a reason to believe there is a difference in incontinence presentation and treatment across genders. Our research strives to understand the influences of gender, obesity, and waist circumference on different types of incontinence.

Methodology

Data were gathered from the Centers for Disease Control and Prevention’s National Health and Nutrition Examination Survey dataset. Questionnaire data from March 2017 through March 2020 categorized as “Kidney Conditions - Urology” and “Weight History” were collected. Binary logistic regressions were performed to examine the association between variables associated with obesity including body mass index (BMI) and waist circumference and if the participant had a urine leak during physical activities. Covariates such as waist circumference, gender, age, race, educational level, and marital status were controlled for.

Results

We found that stress incontinence was positively associated with BMI, waist circumference, and age in men with regression coefficients of 0.038, 0.014, and 0.027, respectively, with a p-value <0.05. In women, stress incontinence was also associated with BMI, waist circumference, and age in addition to being white and being married. Linear regression coefficients were 0.036, 0.019, 0.015, -0.473, and -0.285, respectively, with p-values <0.05.

Conclusions

Our results suggest that BMI, waist circumference, and age are positively correlated with stress incontinence in both men and women. This is consistent with previous literature yet novel in evaluating stress incontinence in men. This would indicate that stress incontinence is similar among men and women which would indicate that weight loss is a therapeutic target for the treatment of stress incontinence in men. However, our findings additionally highlight the correlation between stress incontinence in women and race, a relationship not seen in men. This identifies a possible difference in the pathophysiology of stress incontinence across genders and would require further investigation into therapeutic treatments in men.

## Introduction

Urinary incontinence (UI) is the loss of bladder control and is a common condition found more often in women. Incontinence can present in numerous ways. The various forms of incontinence include urgency urinary incontinence (UUI), stress urinary incontinence (SUI), and mixed urinary incontinence (MUI) (a combination of both SUI and UUI). 

Studies have shown obesity in women to be correlated with a higher prevalence of UI compared to non-obese women [1-3]. A systematic review conducted by Hunskaar et al. found evidence to support that body mass index (BMI) is a risk factor for the development of UI in women [4]. Longitudinal data analyzed by Townsend et al. showed a similar finding. They concluded that BMI was associated with an increased risk of UUI and MUI. However, they found no such association between BMI and SUI when controlling for the effect of waist circumference. They did find that waist circumference alone was associated with the development of SUI and not UUI or MUI [5]. The findings of these studies suggest a possible difference between SUI and other forms of incontinence when evaluating incontinence by subtype. Additionally, there is a limitation to these studies as the analysis is solely regarding incontinence in women.

A similar discrepancy between subtypes emerges when looking at interventions. In a randomized controlled clinical trial conducted by Subak et al., women with an average weight loss of 16 kg saw a reduction in UI episodes compared to those with no weight loss [6]. The Diabetes Prevention Program randomized control trial demonstrated similar findings. The prevalence of episodes of both stress and urge incontinence significantly decreased with a decrease in weight and healthier lifestyle choices [7]. The Program to Reduce Incontinence by Diet and Exercise study reported identical findings to that of the Diabetes Prevention Program regarding the effects of weight loss on stress and urge incontinence. However, a later study by Subak et al. described weight loss to decrease the frequency of stress-incontinence episodes, but not of urge-incontinence episodes indicating there may be a difference in the pathology of the two conditions [8]. Once again, in addition to the difference between subtypes, these studies do not account for the possible differences between men and women.

There have been a few studies that have specifically analyzed SUI in men and described differences. Khullar et al. showed obesity rates were the highest among those with MUI (men and women), SUI in women, and UUI in men. Regression showed that a BMI of 30 kg/m^2^ (obese) was associated with UI in general and MUI in women and UUI in men. However, being overweight was unrelated to any form of UI in men [9]. This indicates a stark comparison between incontinence subtypes across genders. Wolin et al. showed obesity as a risk factor for post-prostatectomy incontinence [10]. Similarly, post-prostatectomy incontinence severity was associated with faster BMI gain after radical prostatectomy, and the magnitude of incontinence improvement after artificial urinary sphincter insertion was associated with greater BMI reduction [11]. However, these studies did not identify incontinence by subtype.

The importance of identifying potential risk factors for various subtypes of incontinence is crucial for targeting appropriate therapeutic interventions. Some studies have previously shown weight loss as an intervention for both stress and urge incontinence in women, as described above. However, given the discrepancy between studies and the lower prevalence of UI in men compared to women, there is a paucity of data on the relationship between BMI and UI in men and the potential of various therapeutic interventions. Demonstrating the relationship between BMI and stress incontinence in men similar to women would indicate that weight loss would be an effective intervention for men as well. In this cross-sectional, observational study using the National Health and Nutrition Examination Survey (NHANES) database, we investigate the relationship between BMI and SUI in men compared to women.

## Materials and methods

Data were gathered from the NHANES dataset from the Centers for Disease Control and Prevention (CDC). NHANES is a program conducted by the CDC to gather information about the health and nutritional status of people in the United States. The survey includes interviews and physical examinations and collects data on a variety of health indicators. Questionnaire data from March 2017 through March 2020 categorized as “Kidney Conditions - Urology” and “Weight History” were collected. The study was approved by the Physician’s Journal of Medicine Review Board, Queens, New York, United States on February 24th, 2023 (approval number: 2302F001). A binomial multivariate logistic regression was used to examine the association between variables associated with obesity, including BMI and waist circumference, and whether the participant had a urine leak during physical activities. Alpha was set to 0.01, and a p-value <0.05 was considered significant. All analyses were performed using SPSS Statistics for Windows, Version 28.0 (IBM Corp., Armonk, NY, United States). BMI and waist circumference were separated into different models due to multicollinearity, and the Akaike information criterion (AIC) was used to compare these models. Lower AIC levels indicate better models and differences >10 were used to indicate that the lower AIC model may be significantly better [12]. Covariates such as waist circumference, gender, age, race, educational level, and marital status were controlled for.

## Results

The NHANES cycle from 2017 to 2020 had 7,955 participants who completed the relevant surveys, of whom 1,970 (25%) reported a history of stress incontinence in the past 12 months and 5,985 (75%) did not. Of the 25% of participants who reported incontinence in the past 12 months, 233 (4%) were male while 1,737 (96%) were female (Table 1). The difference in prevalence is consistent with the literature that stress incontinence is more commonly found in women [13].

**Table 1:** Incidence of stress incontinence by race, gender, marital status, and education level.

Variable	Stress urinary incontinence	No stress urinary incontinence
Gender		
Male	233 (5.97%)	3,673 (94.03%)
Female	1,737 (42.9%)	2,312 (57.1%)
Race/Hispanic origin		
Mexican American	249 (27.07%)	671 (72.93%)
Other Hispanic	199 (24.15%)	625 (75.85%)
White	834 (29.47%)	1,996 (70.53%)
Black	411 (19.48%)	1,699 (80.52%)
Asian	175 (19.71%)	713 (80.29%)
Education level		
Less than 9th grade	129 (22.83%)	436 (77.17%)
9–11th grade	239 (27.92%)	617 (72.08%)
High school graduate/GED or equivalent	471 (24.34%)	1,464 (75.66%)
Some college/AA degree	675 (25.69%)	1,952 (74.31%)
College graduate or above	455 (23.17%)	1,509 (76.83%)
Marital status		
Married	1,113 (24.25%)	3,476 (75.75%)
Widowed/Divorced/Separated	582 (32.28%)	1,221 (67.72%)
Never married	273 (17.56%)	1,282 (82.44%)

Regression analysis of males demonstrated that stress incontinence was associated with increased BMI (coefficient = 0.038, confidence interval (CI) = 0.018 to 0.058, p < 0.001) and age (coefficient = 0.041, CI = 0.031 to 0.050, p < 0.001). The AIC for this regression model was 1,619. When BMI was replaced with waist circumference, stress incontinence was associated with increased waist circumference (coefficient = 0.014, CI = 0.005 to 0.022, p = 0.002) and age (CI = 0.027 to 0.046, p < 0.001). The AIC for this model was 1,584. The difference between these models was 35 indicating that waist circumference is likely a better model to predict stress incontinence based on correlation. However, both models indicated similar findings that stress incontinence in men is positively correlated to obesity and age.

Regression analysis of females demonstrated that stress incontinence was associated with increased BMI (coefficient = 0.036, CI = 0.028 to 0.044, p < 0.001) and age (coefficient = 0.017, CI = 0.013 to 0.021, p < 0.001). It was also associated with being white (coefficient = -0.498, CI = -0.635 to -0.360, p < 0.001) and with being married (coefficient = -0.278, CI = -0.409 to -0.147, p < 0.001). The AIC for this model was 5,190. When BMI was replaced with waist circumference, stress incontinence was associated with increased waist circumference (coefficient = 0.019, CI = 0.015 to 0.022, p < 0.001) and age (coefficient = 0.015, CI = 0.011 to 0.018, p < 0.001). It was also associated with being white (coefficient = -0.473, CI = -0.612 to -0.333, p < 0.001) and being married (coefficient = -0.285, CI = -0.418 to -0.152, p < 0.001). The AIC for this model was 5,026. The difference between these models was an AIC of 164, indicating that the waist circumference model is likely a better model to predict stress incontinence based on correlation. It is important to note here as well as with men that waist circumference is a better model than BMI, with both models showing similar findings. Stress incontinence in women was more likely to be associated with obesity, age, being white, and being married (Table 2).

**Table 2:** BMI and waist circumference by gender AIC = Akaike information criterion; BMI = body mass index

Variables	Odds ratio	Coefficient	95% confidence interval	P-value	AIC
Male					
BMI model	1.039	0.038	0.018 to 0.058	<0.001>	1,619
Waist circumference model	1.014	0.014	0.005 to 0.022	<0.002>	1,584
Female					
BMI model	1.037	0.036	0.028 to 0.044	<0.001>	5,190
Waist circumference model	1.019	0.019	0.015 to 0.022	<0.001>	5,026
Age					
BMI model	1.017	0.017	0.013 to 0.021	<0.001>	
Waist circumference model	1.015	0.015	0.011 to 0.018	<0.001>	
White					
BMI model	0.607	-0.498	-0.635 to -0.360	<0.001>	
Waist circumference model	0.623	-0.473	-0.612 to -0.333	<0.001>	
Married					
BMI model	0.723	-0.323	-0.409 to -0.147	<0.001>	
Waist circumference model	0.751	-0.285	-0.418 to -0.152	<0.001>	

## Discussion

The literature on the correlation between weight/BMI and incontinence subtypes is conflicting. Some studies show that obesity is linked to a higher prevalence of UI, while others find no association between BMI and SUI after controlling for waist circumference [1-4]. Treatment options for incontinence subtypes also differ in the literature. Some studies suggest that weight loss decreases the frequency of episodes of both stress and urge incontinence [6,7], while others find it only affects stress incontinence [8]. Most studies have only been conducted among women and have not differentiated between subtypes. A few studies evaluated male incontinence and found conflicting results [10,11] but did not differentiate between subtypes.

Due to the lack of data and clear conflict in the literature, our novel study aimed to further understand the relationship between gender, BMI, and stress incontinence.

Our results indicate that stress incontinence in both men and women is positively correlated with obesity and age whether looking at BMI or waist circumference and that linear regression models show more robust results favoring waist circumference as a predicting factor. This is consistent with previous literature that female incontinence is correlated with BMI [1-4]. However, this is still a novel finding considering the unique look at collinearity and the comparison of waist circumference and BMI. Additionally, we focused on comparing males to females and focused on stress incontinence which had conflicting literature [5,9].

Our results also indicate an association between female stress incontinence related to being white and married whereas male stress incontinence shows no such association. This may be explained by the increased prevalence of stress incontinence in women following pregnancy [14,15]. However, the association of race may indicate a difference in the pathophysiology of stress incontinence between men and women.

These findings indicate that weight loss may be a potential therapeutic target for men with stress incontinence consistent with the literature on females [6-8]. However, this would require further investigation as it is unclear if weight loss would be therapeutic for men given that there are discrepancies among treatment and presentation across genders [9].

There are several limitations to this study that should be acknowledged. First, the sample size was relatively small and may not be representative of the broader population. Second, the study relied on self-reported data, which is subject to biases and may not be entirely accurate. Third, as a cross-sectional study, it cannot establish causality; therefore, it is difficult to determine the direction of the relationship between the variables.

## Conclusions

Our study found that BMI, waist circumference, and age were positively correlated with SUI in both men and women. This is consistent with previous literature, but our findings are novel in that we identified stress incontinence specifically in men. Therefore, similar therapeutic interventions, such as weight loss, should be considered in both men and women with stress incontinence. However, our findings also revealed the correlation of stress incontinence in women with race, a relationship not seen in men. This suggests a possible difference in the pathophysiology of stress incontinence across genders and highlights the need for further investigation into therapeutic treatments in men. Our study supports the importance of considering both gender and incontinence subtype when evaluating risk factors and designing therapeutic interventions for UI.
